# A Lactic Acid Bacteria (LAB)-Based Vaccine Candidate for Human Norovirus

**DOI:** 10.3390/v11030213

**Published:** 2019-03-02

**Authors:** Kelsey Craig, Xianjun Dai, Anzhong Li, Mijia Lu, Miaoge Xue, Lucia Rosas, Thomas Z. Gao, Andrew Niehaus, Ryan Jennings, Jianrong Li

**Affiliations:** 1 Department of Veterinary Biosciences, College of Veterinary Medicine, The Ohio State University, Columbus, OH 43210, USA; Craig.371@osu.edu (K.C.); xjdai@cjlu.edu.cn (X.D.); li.7875@osu.edu (A.L.); lu.1383@osu.edu (M.L.); xue.303@osu.edu (M.X.); rosas.3@osu.edu (L.R.); gao.935@osu.edu (T.Z.G.); jennings.398@osu.edu (R.J.); 2 Department of Veterinary Clinical Sciences, College of Veterinary Medicine, The Ohio State University, Columbus, OH 43210, USA; Niehaus.25@osu.edu

**Keywords:** human norovirus, lactic acid bacteria, gnotobiotic piglet, vaccine, VP1

## Abstract

Human noroviruses (HuNoVs) are responsible for more than 95% of the non-bacterial acute gastroenteritis epidemics in the world. The CDC estimates that every year 21 million individuals suffer from HuNoV-induced gastroenteritis in the United States. Currently, there is no FDA-approved vaccine for HuNoVs. Development of an effective vaccine has been hampered by the lack of an efficient cell culture system for HuNoVs and a suitable small animal model for pathogenesis study. In this study, we developed lactic acid bacteria (LAB) as a vector to deliver HuNoV antigen. A LAB strain (*Lactococcus lactis*) carrying VP1 gene of a HuNoV GII.4 virus (LAB-VP1) was constructed. It was found that HuNoV VP1 protein was highly expressed by LAB vector and was secreted into media supernatants. To test whether LAB-based HuNoV vaccine candidate is immunogenic, 4-day-old gnotobiotic piglets were orally inoculated with various doses of LAB-VP1. It was found that LABs were persistent in the small intestine of piglets and shed in pig feces for at least 25 days post inoculation. LAB DNA and VP1 were detected in mesenteric lymph nodes and spleen tissue in LAB-VP1 inoculated groups. HuNoV-specific IgG and IgA were detectable in serum and feces respectively at day 13 post-inoculation, and further increased at later time points. After being challenged with HuNoV GII.4 strain, a large amount of HuNoV antigens were observed in the duodenum, jejunum, and ileum sections of the intestine in the LAB control group. In contrast, significantly less, or no, HuNoV antigens were detected in the LAB-VP1 immunized groups. Collectively, these results demonstrate that a LAB-based HuNoV vaccine induces protective immunity in gnotobiotic piglets.

## 1. Introduction

Human norovirus (HuNoV) is the causative agent of more than 95% of nonbacterial acute gastroenteritis cases worldwide [1]. HuNoV is responsible for over 60% of foodborne illnesses in the United States. It is estimated that 21 million people are infected by HuNoV, which result in approximately 800 deaths annually in the United States. HuNoV causes a global economic burden, with $60.3 billion in healthcare costs on a yearly basis [2,3]. HuNoV is highly infectious and contagious, and approximately ten virus particles are sufficient to establish an infection [4,5]. However, research on HuNoV has been hampered because it cannot be efficiently grown in cell culture and lacks a suitable small animal model. Currently, HuNoV is listed as a “candidate contaminant” for the regulation of drinking water by EPA and is classified as a Category B Priority Pathogen by the National Institute of Allergy and Infectious Diseases [6]. To date, there is no FDA approved vaccine or antiviral drug to combat HuNoV. Vaccination is the most effective strategy to prevent infectious diseases. A HuNoV vaccine is urgently needed to protect the population, particularly for the most susceptible populations, including infants, children, the elderly, and immunocompromised individuals [2].

Since the discovery of HuNoV in 1968, major efforts have been devoted to developing a safe and efficacious HuNoV vaccine. Currently, most HuNoV vaccine studies have been focused on a subunit vaccine using the viral capsid protein (VP1) as the antigen. Expression of the VP1 gene in insect cells can lead to the subsequent self-assembly of virus-like particles (VLPs) that are structurally and antigenically similar to native virions. It has been shown that VLP-based vaccines trigger a variable level of HuNoV-specific serum antibody response and mucosal immunity in mice, rabbits, guinea pigs, gnotobiotic piglets, and chimpanzees [7,8,9,10]. The VLP-based vaccine candidate has been tested in human clinical trials. In 1999, Ball et al. demonstrated that human norovirus VLPs were safe and immunogenic in a human clinical trial study [11]. Over the last 18 years, there have been multiple phase I and II clinical trials of numerous VLP-based vaccines to assess the protection and immune response potential. A recent human clinical trial showed that the Norwalk virus-specific IgA antibody was detected in 70% of the vaccine recipients. After being challenged with the Norwalk virus, it was found that vaccination significantly reduced the frequency of Norwalk virus gastroenteritis. Of the placebo participants, 67% developed gastroenteritis, whereas only 37% of vaccine recipients developed symptoms [12]. In another double-blind, placebo-controlled trial, 18- to 50-year-olds received 2 injections of norovirus GI.1/GII.4 bivalent VLP vaccine with 3-O-desacyl-4′-monophosphoryl lipid A (MPL), and alum significantly reduced norovirus-related vomiting and diarrhea [13]. Despite the promising results of these studies, production of VLPs is time-consuming and expensive, and immunization requires high VLP doses, multiple boosters, and mucosal adjuvants, such as *V. cholerae* and *E. coli* enterotoxins [7]. In addition, immunogenicity of such vaccines is limited, as the VLPs are non-replicating immunogens.

Since a conventional live attenuated vaccine cannot be developed from a virus that cannot be efficiently propagated in vitro, several viral vectors have been explored to deliver HuNoV vaccine candidates. These viral vectors include Venezuelan equine encephalitis (VEE), adenovirus, vesicular stomatitis virus (VSV), and Newcastle disease virus (NDV) [14,15,16,17]. Mice immunized with these viral vectored vaccine candidates triggered strong HuNoV-specific immunities [14,15,16,17]. Whether these viral vectored vaccine candidates are protective is unknown. In addition, the safety concern of these viral vectors limited their practical application in humans. Recently, Jones et al. reported that HuNoV is capable of replicating in human B cells, and that commensal bacteria (such as *Enterobacter cloacae*) facilitates such replication [18]. Controversially, other researchers found that *Enterobacter cloacae* inhibited human norovirus infectivity in gnotobiotic pigs [19]. Ettayebi et al. also reported that multiple HuNoV strains can replicate in stem cell-derived human enteroids [20]. Although these studies are highly promising, it is unknown whether HuNoV can continuously be passed in these cell culture systems to develop a live attenuated HuNoV vaccine.

A live bacteria delivery system offers enormous potential for the development of new vaccines against infectious diseases. However, this strategy has not been explored in HuNoV vaccine development. Food grade lactic acid bacteria (LAB) are an excellent platform to fulfill this requirement. Food grade LAB are an attractive delivery system, as they are non-pathogenic, effective in delivering antigens to the mucosa, and FDA approved GRAS (Generally Recognized As Safe) agents. Several species of *lactobacilli* and *lactococci* are known to be excellent vehicles for delivery of vaccines against a spectrum of infectious agents, including HIV, rotavirus, human papillomavirus, porcine circovirus type 2 (PCV2), *Streptococcus pneumoniae*, *Clostridium tetani*, *Brucella abortus*, *Rhodococcus equi*, and *Staphylococcus aureus* [21,22,23,24,25,26,27]. *Lactococcus lactis* is a gram-positive lactic acid producing bacterium commonly used in the dairy industry. In addition to its high safety profile, oral vaccination of mice with *Lactococcus lactis* vectored vaccine induced a strong systemic immune response and mucosal immune response. Although it has not been licensed for use in humans, preclinical studies showed that LAB-based vaccine is promising for future development. This vaccine strategy is particularly attractive for HuNoV, as an ideal HuNoV vaccine must be safe, stable, inexpensive, easy to deliver, and able to induce robust humoral, mucosal, and cellular immune responses at sites where pathogens interact with the host.

In this study, we developed a LAB-based HuNoV vaccine candidate. The major capsid gene (VP1) of a GII.4 HuNoV strain was cloned into a LAB expression vector pNZ8150, which was subsequently transformed into *Lactococcus lactis* by electroporation, resulting in a LAB bacteria strain expressing VP1 (LAB-VP1). Subsequently, we showed that HuNoV VP1 protein was highly expressed by LAB vector, and the expressed VP1 was secreted into media supernatants. Oral vaccination of LAB-VP1 in gnotobiotic piglets triggered HuNoV-specific IgA, and IgG responses and prevented HuNoV infection of pig intestines. Collectively, these results demonstrate that LAB-based HuNoV vaccine is immunogenic in gnotobiotic piglets. Our results also suggest that a LAB-based HuNoV vaccine is a promising vaccine candidate for HuNoV.

## 2. Materials and Methods

### 2.1. Preparation of Human Norovirus Inoculum

The HuNoV GII.4 strain 766 was kindly provided by John Hughes (College of Medicine, The Ohio State University). Stool samples were diluted 1:2 in minimal essential medium (MEM; Gibco-Invitrogen, Carlsbad, CA) and further processed by vortexing, centrifugation at 3500 × *g* for 20 min, and filtration through a 0.8-μm-pore-size filter, followed by a 0.2-μm-pore-size filter. The possibility of the presence of other enteric viral pathogens, such as human rotavirus, human sapovirus, and human astrovirus, was excluded by RT-PCR analysis prior to initiation of the study. The amount of RNA copies in the HuNoV strain 766 filtrate was quantified by real-time RT-PCR, and the level of RNA was 2.1 × 10^8^ RNA copies/mL. Viruses were aliquoted and stored at −80 °C until use.

### 2.2. Bacterial Cultures

The Nisin controlled gene expression (NICE) system strain NZ9000 *Lactococcus lactis subsp*. *Cremoris*, containing regulatory genes nisR and nisK integrated into the pepN gene, was used as a vector for HuNoV. The ORF of VP1 gene of HuNoV GII.4 strain 766 was amplified by RT-PCR then cloned into pNZ8150 NICE expression secretion vector (MoBiTec), which contains a chloramphenicol resistance gene using cloning site NaeI resulting in a pNZ8150- GII.4 VP1 vector. This pNZ8150- GII.4 VP1 vector was transformed into *Lactococcus lactis* via electroporation providing lactic acid bacteria (LAB) capable of expressing GII.4 VP1 protein, named LAB-VP1. In addition, the empty pNZ8150 vector was transformed into *Lactococcus lactis* to use for control purposes. This control LAB was named LAB empty vector control. For the culturing of LAB-VP1 and LAB empty control, M17 medium and agar containing 1% (*wt*/*vol*) glucose and 10 µg/mL chloramphenicol were used, along with a GasPak anaerobic chamber providing optimal anaerobic growing conditions for the LAB-VP1 to grow overnight at 30 °C. For long-term storage of LAB-VP1, M17 medium containing 25% glycerol was aliquoted and stored at −80 °C.

### 2.3. Protein Expression

The expression of HuNoV VP1 protein was detected using Western blot analysis of the supernatant and cell lysate. A single clone of LAB-VP1 or LAB control was grown in M17 medium overnight then diluted 1/25 in 2 tubes with 10 mL of M17 for it to grow for another 4–5 h until an OD600 of 0.4 was reached. One of the 10 mL tubes was induced with 2 ng/mL Nisin, and the second tube served as control. The LAB-VP1 was incubated for another 5 h then the supernatant and cells were harvested separately by centrifugation at 4000 rpm for 10 min at room temperature. The cells were resuspended in 300 µL of lysate buffer (50 mM Tris-HCl, 2 mM EDTA, 10 mM NaCl, 0.1% Triton X-100, pH 8) and 10mg/mL lysozyme and incubated at 37 °C for 3 h then ultrasonicated 3 times for 40 s. The supernatant was ultracentrifuged at 30,000 rpm for 2 h and the pellet was resuspended in lysate buffer. The VP1 protein was analyzed by Western blot analysis. The cell lysate and supernatant from Nisin and the control samples were prepared by adding 5× loading buffer and boiling for 7 min. For SDS-PAGE, the samples were added to a 10% acrylamide gel then run at 80 V for 30 min then 120 V for 1 h. The gel was then transferred to a Hybond enhanced chemiluminescence nitrocellulose membrane (GE Healthcare Bio-Sciences, Pittsburgh, PA, USA) via a Mini Trans-Blot electrophoretic transfer cell (Bio-Rad, Hercules, CA, USA). The primary antibody guinea pig anti-HuNoV VP1 antiserum (a generous gift from Dr. Xi Jiang, Cincinnati Children’s Hospital) was diluted 1:5000 in blocking buffer (5% non-fat milk) followed by horseradish peroxidase-conjugated goat anti-guinea pig IgG secondary antibody (Santa Cruz Biotechnology, Dallas, TX, USA) at a dilution of 1:10,000. The blot was developed via a SuperSignal West Pico Chemiluminescent Substrate (Thermo Scientific, Waltham, MA, USA) and exposed to Kodak BioMax MR film (Kodak, Rochester, NY, USA).

### 2.4. The stability of L. Lactis in pH Differences

The stability of LAB-VP1 was assessed at pH 2, 4, 7, and 8.5 at 37 °C and pH 7 at 4 °C at differing time points. The LAB-VP1 was grown overnight as described above, then undiluted, diluted 10 (1:10) times, and diluted 100 (1:100) times samples were centrifuged for 1 min at 4500 rpm at room temperature. For the 10 times concentrated samples, LAB-VP1 was centrifuged 4000 rpm for 10 min at room temperature. Then, saline buffer with different pH was added and the mixture was aliquoted by adding 0.5 mL per tube and placed at 37 °C. For stability assay at pH 2, the undiluted and 10 × concentrated tubes were removed at 30, 60, 90, and 120 min. The diluted 1:10 and 1:100 tubes (pH 2) were removed at 3, 6, 9, 12, 15, and 18 min. The pH 7 and pH 8.5 tubes were removed at 12, 24, 36, and 48 h. The pH 4 tubes were removed at 4, 8, and 12 h. For stability assay at pH 7 at 4 °C, tubes were removed at 3, 7, 10, 14, 17, 21, 24, and 28 days. The pH reaction was stopped by centrifuging 4500 rpm for 1 min at room temperature then 1 mL of M17 medium was added and vortexed to resuspend the bacteria. The survival was calculated by a 10-fold serial dilution with saline then plating of several dilutions on M17 plates for each time point. After 2 days, the colony forming units (cfu) were quantified to determine the original titer at the assigned time points.

### 2.5. Delivery of Gnotobiotic Piglets

The animal protocol used in this study followed the USDA regulations and guidelines of Guide for the Care and Use of Laboratory Animals of the National Institutes of Health. Also, the animal protocol was approved by The Ohio State University Institutional Laboratory Animal Care and Use Committee (animal protocol no. 2015A00000118). Two adult artificially inseminated pregnant Landrace sows were purchased from a commercial pork production unit (Shoup Brothers, Smithville, OH, USA) and transported to the Goss Laboratory at The Ohio State University. The sows were on their 113th day of gestation and provided 21 and 19 piglets, respectively. The sows were administered 20 cc of 2% lidocaine in the epidural space at the lumbosacral junction. Effect of the epidural was assessed by the loss of motor function of the rear legs. Following epidural administration of lidocaine, a mixture of ketamine and telazol was given intramuscularly 10 min prior to the surgery. After recumbency was achieved, the sow was lifted onto a surgery table and an aseptic prep was performed. A paramedian approach was made into the abdomen and the entire gravid uterus was exteriorized, and a closed hysterectomy was performed. The uterus was transferred through a bleach water tunnel into a surgical isolator. The piglets were removed from the uterus in a sterile environment, and transferred through a plastic sleeve into an attached rearing isolator in order to apply umbilical clamps, resuscitate, and dry them with sterile towels. Only 25 piglets survived due to some piglets exhibiting severe hypoglycemia overnight. The rearing isolator was closed and detached, then the piglets were separately housed in sterile isolators made of vinyl canopies connected with 6 partitions. Insulators were maintained under positive pressure via 2 AW-40 filters for the entirety of the experiments. The derivation units, 4 isolators and supplies were sterilized via sporklenz (peracetic acid, hydrogen peroxide, and acetic).

For an external heat source, several heat lamps were stationed around the isolators to keep the temperature at 33–34 °C for the first week, and reduced over a three-week period to 23 °C. The piglets were fed Natrel whole milk three times a day in a weekly increasing volume schedule. On the first day the piglets received vitamin B complex and Iron intramuscularly (IM). Contamination was checked 3 days prior to derivation and 1 day before euthanasia. Sterile swabs (8/isolator) were used to pick up contamination in each isolator. Four of the swabs were streaked on sheep blood agar plates and four of the swabs per isolator were placed in soy broth. The plates were placed in aerobic and anaerobic conditions at 37 °C along with the soy broth. The plates and broth were checked for contamination at 24, 48, and 72 h.

### 2.6. Immunogenicity of LAB-VP1 Vaccine Candidate in Gnotobiotic Piglets

**Figure 1:**
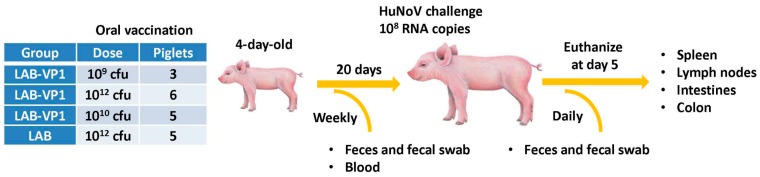
Flow diagram of gnotobiotic piglet experimental design.

Experimental design was depicted in Figure 1. LAB-VP1 and LAB were grown, quantified, and stored at 4 °C for 2 days prior to use. Briefly, four-day-old newborn gnotobiotic piglets were inoculated orally via oral gavage with 10 mL of LAB-VP1 at three different doses, 10^9^ cfu, 10^10^ cfu, and 10^12^ cfu, and LAB vector control at 10^12^ cfu in saline. Before inoculation blood, fecal, nasal, and vaginal swabs were taken as controls. At days 6, 13, and 20 post inoculation, blood, fecal, nasal and vaginal swabs were collected. The blood samples were centrifuged at 10,000 rpm for 2 min to separate the sera, which were used for detection of HuNoV-specific IgG by ELISA. The fecal, nasal, and vaginal swabs were used for the detection of HuNoV-specific IgA by ELISA. The other half of the fecal swabs was used to enumerate the *L. lactis*. At day 20 post-inoculation, the piglets were challenged with 1.0 × 10^7^ genomic RNA copies of HuNoV GII.4 strain 766 diluted in 5 mL of saline, given via oral gavage. Over the following 4 days, fecal samples were collected to quantify the RNA copies of HuNoV. At day 5 post-challenge, the piglets were euthanized and necropsied. The spleen, mesenteric lymph nodes, intestinal pieces, jejunum content, blood, and colon content were taken to quantify *L. lactis* by plating, quantify HuNoV RNA by real-time RT-PCR, detect HuNoV antigen expression by immunofluorescence assay (IFA), and determine the IgA or IgG antibody titers.

### 2.7. Quantification of LAB Titer in Pig Tissues and LAB Shedding in Pig Feces

The intestinal pieces from the duodenum, jejunum, and ileum were placed in 2 mL of saline and weighed prior to processing. Each piece was scraped with a scalpel on a petri dish then 10-fold serial diluted in saline and plated on M17 plates as described previously. The colon and jejunum content were weighted then diluted with saline (2 × *vol*/*wt*, then serially diluted and plated as previously described. The mesenteric lymph nodes and spleen were weighted first, then homogenized with 15 mL dunces in 3 mL saline, then serially diluted and plated as described previously. All experiments were done aseptically to avoid cross-contamination.

### 2.8. Detection of HuNoV RNA by Real-Time RT-PCR

The piglet fecal samples were eluted in 300 µL of DMEM at a 2:1 (*vol*/*wt*) dilution. The fecal samples were vortexed then centrifuged at 10,000 rpm for 15 min at 4 °C. The supernatants were collected to extract RNA with the RNeasy Mini kit (Qiagen, Valencia, CA, USA) and eluted in 30 µL of ultrapure water. The HuNoV RNA was detected and quantified by real time RT-PCR using primers annealing to RNA-dependent RNA polymerase (RdRp) gene of HuNoV, as this region is relatively conserved in the HuNoV genome. The first-strand cDNA was synthesized using SuperScriptase III (Invitrogen, Carlsbad, CA, USA), using a RdRp reverse primer (5′- ACCACGCTAGGAGAAAGAAGGTC-3′) in addition to 10 mM dNTP, 5 µL of RNA template, 1 unit of SuperScriptase III, 5× first strand buffer, and 0.1M DTT. To amplify the RdRp gene, a combination of SYBR Green Master Mix (Takara, Berkeley, CA, USA), ROX, forward primer (5′-AGTTGGCATGAATATGAATGAGGA-3′), and reverse primer (5′ ACCACGCTAGGAGAAAGAAGGTC-3′) with 5 µL of cDNA template were placed in a StepOne real-time PCR machine (Applied Biosystems, Foster City, CA, USA). For each cycle, a holding stage at 95 °C was maintained for 2 min prior to cycling, followed by 40 cycles of 94 °C for 15 s for denaturation, 55 °C for 30 s for annealing, and 72 °C for 15 s for extension. The sample CT values were analyzed with the HuNoV RdRp plasmid of known concentration to calculate the log_10_ RNA copies/g.

### 2.9. Indirect Immunofluorescence Assay (IFA) on Whole Intestinal Tissue Mounts

Indirect immunofluorescence was performed on whole-mount intestinal tissues. Pieces of duodenum, jejunum, and ileum from inoculated pigs were collected and fixed with 2 mL of 4% paraformaldehyde-0.2% glutaraldehyde in 0.1 M potassium phosphate buffer (PPB) (pH 7.4) for 2 h at RT. The fixed samples were washed four times with PPB, quenched with 1 mL of PPB containing 50 mM glycine, and stored at 4 °C. After quenching overnight at 4 °C, sections of the tissues were cut and permeabilized with 0.3 mL of 0.1% Triton X-100 in PBS for 1 h then washed 3 times with PBS. Then, the tissues were blocked with 0.5 mL PBS containing 2% bovine serum albumin and 5% goat serum for 1.5 h at RT. The tissues were incubated with guinea pig anti-HuNoV at a 1:5000 dilution overnight at 4 °C in 0.3 mL of incubation buffer containing 10 mM potassium phosphate buffer (PPB) [pH 7.4], 150 mM NaCl, 10 mM sodium azide, and 0.2% bovine serum albumin. After washing them with PBS six times, the tissues were incubated with the secondary antibody goat anti-guinea pig IgG (Invitrogen; A11075) labeled with AlexaFluor488 [Ex (nm) 499, Em (nm) 519], which produces a green color at a dilution of 1:800 in 0.3 mL of incubation buffer. The tissues were then stained with the nuclear stain SYTOX orange (Invitrogen; S11368) [Ex (nm) 547, Em (nm) 570] diluted 1:1000 in PBS for 15 min on a shaker, giving a red color. Then, the actin was stained with AlexaFluor633-labeled 149 phalloidin (Invitrogen; A222884) [Ex (nm) 632, Em (nm) 648] diluted 1:20 for 45 min on a shaker, producing a blue color. Samples were examined using a laser scanning confocal microscope (Olympus FV-1000, Hamburg, Germany) at the Microscopy Facility at The Ohio State University. IFA signal was scored for duodenum, jejunum, and ileum section at ×10 magnification based on the following criteria: score 0 = no IFA signal was observed in villi; score 1 = a small amount of IFA signal was observed in villi; score 2 = a moderate level of IFA signal was observed in villi; and score 3 = a large amount of IFA signal was observed in villi. Average score for each group was shown.

### 2.10. Serum IgG ELISA

The serum samples collected at post inoculation day (PID) 6, 13, 20, and post challenge day (PCD) 5 were used to detect HuNoV-specific IgG. Ninety-six-well plates were coated with 50 μL of highly purified HuNoV VP1 protruding domain particles (P particles) at 20 μg/mL concentration in 50 mM NaCO3 buffer (pH 9.6) at 4 °C overnight. The plates were blocked to prevent nonspecific-protein binding via 0.2 mL per well of 1% (weight/vol) bovine serum albumin (BSA) in PBS-Tween (0.05%) for 2 h at 37 °C. Serum samples were 2-fold-serially diluted with 0.5% BSA in PBST and 50 µL per well of each dilution was added to P particle-coated wells. After incubation at 37 °C for 2 h, the plates were washed three times with PBST, followed by incubation with 100 μL of rabbit anti-swine IgG horseradish peroxidase (HRP) conjugated secondary antibody (Sigma) at a dilution of 1:500 for 1 h at room temperature (RT). Plates were washed three times with PBST then developed with 100 μL of 3′3′,5′5′-tetramethylbenzidine (TMB). The reaction was stopped with 100 µL of 2M sulfuric acid and the optical density (OD) at 450 nm was determined using an enzyme-linked immunosorbent assay (ELISA) plate reader. End point titer values were determined as the reciprocal of the highest dilution that had an absorbance value greater than background level from the LAB empty vector control.

### 2.11. Fecal IgA ELISA

For the fecal samples, HuNoV-specific IgA was determined in PID 6, 13, 20, and PCD5 samples via plates coated with P-particles. Fecal pellets were diluted 2:1 (*vol*/*wt*) in PBS containing 0.1% 150 Tween and a Complete EDTA-free protease inhibitor cocktail tablet (Roche). Samples were vortexed twice for 30 s with 5 min on ice in between then centrifuged at 10,000 × *g* for 10 min. The supernatant was removed to a new tube then centrifuged again. Ninety-six-well plates were coated with 50 μL of highly purified HuNoV P particles at 15 μg/mL concentration in 50 mM NaCO3 buffer (pH 9.6) at 4 °C overnight. For blocking, the plates were incubated for 2 h at 37 °C with 0.2 mL of 1% (weight/vol) BSA in PBST. The samples were 2-fold serially diluted with 0.5% BSA in PBST and 50 µL per well of each dilution was added to P-particle coated plates. The plates were incubated for 2 h at 37 °C then washed 3 times. The secondary antibody goat anti-swine IgA HRP conjugated was added at 1:1,000 diluted in 0.5% BSA in PBST incubated 1h at RT. Plates were washed three times with PBST and developed with 100 μL of 3′3′,5′5′-tetramethylbenzidine (TMB) then the reaction was stopped with 100 µL of 2 M sulfuric acid. The optical density (OD) at 450 nm was determined using an ELISA plate reader. End point titer values were determined as the reciprocal of the highest dilution that had an absorbance value greater than background level from the LAB empty vector control.

### 2.12. Quantification of LAB DNA in Spleen and Lymph Nodes by Real-Time PCR

Total DNA was extracted from sections of the spleen and mesenteric lymph nodes collected at necropsy to determine the amount of LAB DNA. The spleen and lymph nodes were weighed then processed according to the directions in the DNeasy Blood and Tissue kit (Qiagen, Valencia, CA, USA). The DNA was eluted in 30 µL of water. The LAB DNA was quantified by real-time qPCR using primers and a probe designed by Applied Biosystems and the Taqman Fast Universal PCR Master Mix. The real time qPCR procedure was done as described above. The original amount of LAB vector DNA was calculated based on a standard curve using the CT values generated.

### 2.13. Detection of HuNoV VP1 Gene in Spleen and Lymph Nodes by PCR

To determine whether the VP1 gene presents in the spleen and lymph nodes, the DNA was subjected to traditional PCR using the Platinum Blue PCR SuperMix (Invitrogen) with two primers annealing to VP1 gene, forward primer (5′-ATGAAGATGGCGTCGAATGAC-3′), and reverse (5′-TTATAATACACGTCTGCGCCC-3′). For the PCR, a holding stage at 95 °C was maintained for 1 min prior to cycling. For denaturation, 40 cycles at 94 °C for 20 s, 58 °C for 20 s for annealing, and 72 °C for 2 min for extension were done on the samples. The PCR product was run on gel electrophoresis to visualize the presence of HuNoV VP1 gene.

### 2.14. Statistical Analysis

All values are expressed as the means ± standard deviations. Statistical analysis by two-tailed student’s *t*-test was performed. A *p* value of < 0.05 was considered statistically significant.

## 3. Results

### 3.1. HuNoV VP1 Protein is Highly Expressed by LAB Vector

**Figure 2:**
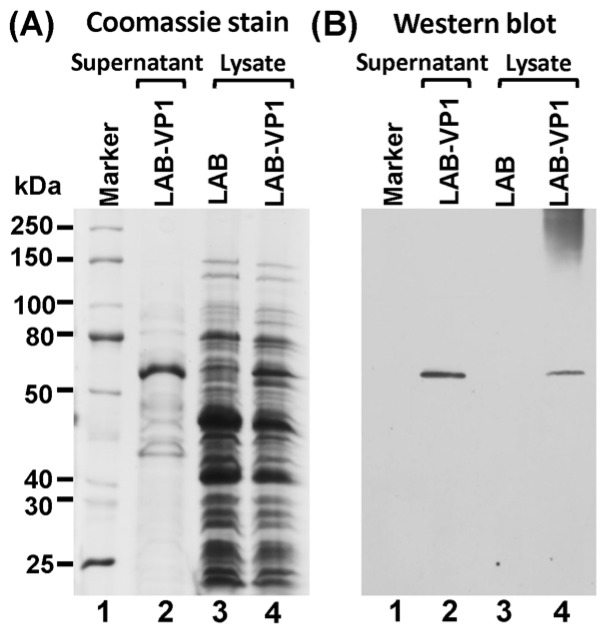
Expression of HuNoV VP1 by LAB vector. LAB-VP1 strain was grown to mid-log phase (O.D. = 0.4) in M17 medium and centrifuged. The cell pellets and supernatants were collected. Proteins from supernatants were precipitated using trichloroacetic acid (TCA) and pelleted by centrifugation. The total protein (10 μg) from both the supernatant and cell pellet was analyzed by (**A**) SDS and PAGE and (**B**) Western blot using HuNoV VP1-specific antibody.

We constructed a recombinant lactic acid bacteria (LAB) expressing HuNoV VP1. The VP1 gene of HuNoV GII.4 strain 766 was amplified by RT-PCR and cloned into the LAB expression vector pNZ8150, which was subsequently transformed into *Lactococcus lactis* by electroporation, resulting in LAB bacteria strains designated “LAB-VP1.” The expression of VP1 protein by the LAB vector was detected by Western blot. Briefly, LAB-VP1 were grown to mid-log phase (O.D. = 0.4) in M17 broth. The cell pellets and supernatants were collected. Proteins from supernatants were precipitated using trichloroacetic acid (TCA) and pelleted by ultracentrifugation. The total protein from both the supernatant and cell pellet was analyzed by SDS-PAGE. As shown in Figure 2A, a 55 kDa protein, consistent with the size of HuNoV VP1, was found in bacteria culture supernatants (Figure 2A, lane 2). To further confirm this, Western blots were performed using anti-HuNoV VP1 polyclonal antibody. VP1 protein was found in both the supernatant (Figure 2B, lane 2) and cell pellet (Figure 2B, lane 4) from LAB-VP1, but not control LAB (Figure 2B, lane 3). Collectively, these data confirm that: (i) HuNoV-VP1 protein is expressed by the LAB vector and, (ii) expressed VP1 protein is secreted into media supernatants.

### 3.2. Stability of LAB-VP1 in Neutral pH Saline Solutions

**Figure 3:**
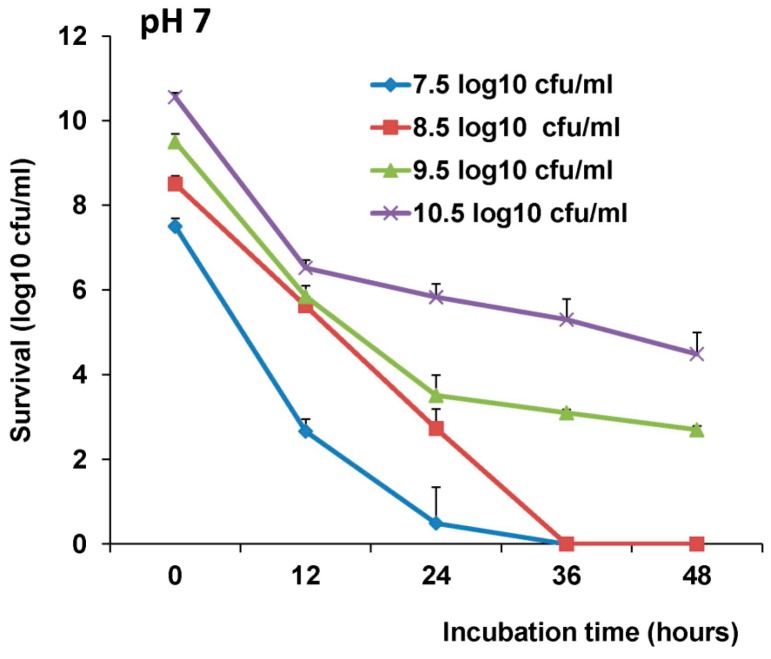
The effects of bacteria concentration and pH 7 on survival of LAB-VP1. The survival of LAB-VP1 at pH 7 solution. The concentrated 10×, undiluted, and diluted 1:100 LAB-VP1 were incubated in pH 7 saline solution. The tubes of each concentration were removed at 12, 24, 36, and 48 h, and bacterial survival was determined by plate counts.

The stability of LAB-VP1 in acidic and basic solutions was assessed to provide insight to the survival in the digestive system. We first examined the stability of LAB-VP1 in neutral pH (pH 7) saline. Briefly, 10 times concentrated (10^10.5^ cfu/mL), undiluted (10^9.5^ cfu/mL), diluted 1:10 (10^8.5^ cfu/mL), and diluted 1:100 (10^7.5^ cfu/mL) LAB-VP1 were resuspended in pH 7 saline and incubated at 37 °C for 12, 24, 36, and 48 h. The reaction was neutralized with M17 medium, and the bacterial titer was determined. As shown in Figure 3, a 3.7 log reduction in undiluted LAB-VP1 titer was observed after 12 h incubation at neutral pH and a 6.0–6.8 log reduction was observed after 24–48 h incubation. Interestingly, when LAB-VP1 was concentrated 10 times, the stability was significantly enhanced at 12 and 24 h (*p* < 0.05). In contrast, LAB-VP1 survival was significantly reduced when LAB-VP1 was diluted 10 and 100 times (*p* < 0.05). All bacteria were inactivated after 36 h incubation at these two dilutions. These results suggested that survival of LAB-VP1 at neutral pH is concentration-dependent.

### 3.3. Stability of LAB-VP1 in pH 8.5 Saline Solutions

**Figure 4:**
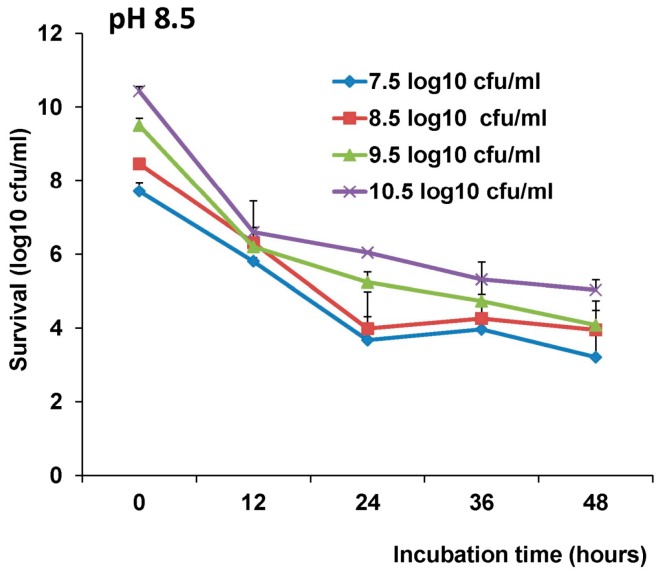
The effects of bacteria concentration and pH 8.5 on survival of LAB-VP1. The survival of LAB-VP1 at pH 8.5 solution. The LAB-VP1 concentrated 10×, undiluted, diluted 1:10, and diluted 1:100 were placed in pH 8.5 saline solution. The tubes of each concentration were removed at 12, 24, 36, and 48 h, and bacterial survival was determined by plate counting.

We next measured the stability of LAB-VP1 at pH 8.5, mimicking the pH environment in the duodenum. For undiluted group, approximately 3.4, 4.2, 4.9, and 5.6 log bacteria reductions were observed after 12, 24, 36, and 48 h incubation (Figure 4). Similarly, survival of LAB-VP1 was increased when it was concentrated 10 times. However, survival was decreased when it was diluted 10 and 100 times (Figure 4). These results demonstrated that LAB-VP1 was more stable at pH 8.5 compared to pH 7.0 (compare log reductions in Figure 3; Figure 4). In addition, these results suggested that the concentration of LAB-VP1 affected the survival of bacteria at pH of 8.5.

### 3.4. Stability of LAB-VP1 in pH 4 Saline Solutions

**Figure 5:**
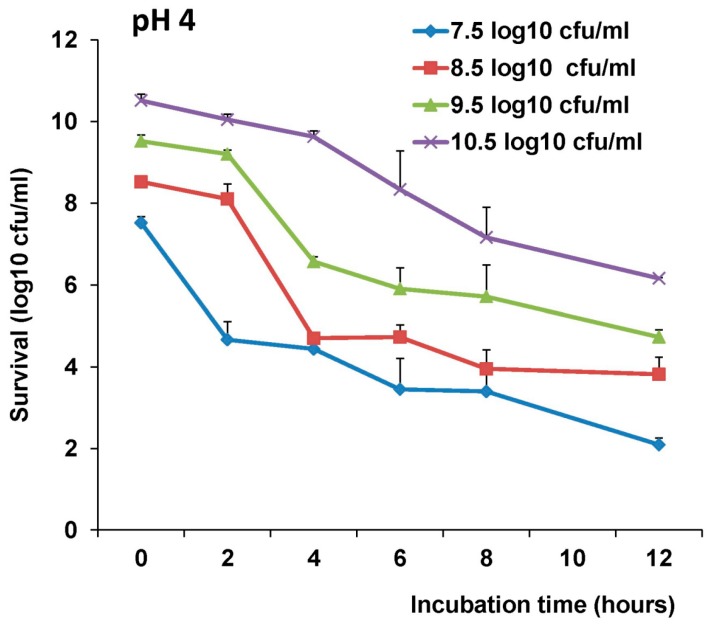
The effects of bacteria concentration and pH 4 on survival of LAB-VP1. The survival of LAB-VP1 concentrated 10×, undiluted, diluted 1:10, and diluted 1:100 after being placed in pH 4 saline solution. The tubes of each concentration were removed at 2, 4, 6, 8, and 12 h.

We next determined the survival of 10 × concentrated, undiluted, diluted 1:10, and diluted 1:100 LAB-VP1 in pH 4 saline with a shorter incubation time, harvesting samples at 2, 4, 6, 8, and 12 h. For undiluted groups, as shown in Figure 5, 0.32, 3.0, 3.6, 3.8, and 4.8 log bacterial reductions were found after 2, 4, 6, 8, and 12h incubation, respectively. Similar to the previous observation in other pH conditions, bacterial survival was significantly enhanced when they were concentrated 10 times (*p* < 0.05), whereas the survival was reduced when they were diluted 10 and 100 times (*p* < 0.05).

### 3.5. Stability of LAB-VP1 in pH 2 Saline Solutions

**Figure 6:**
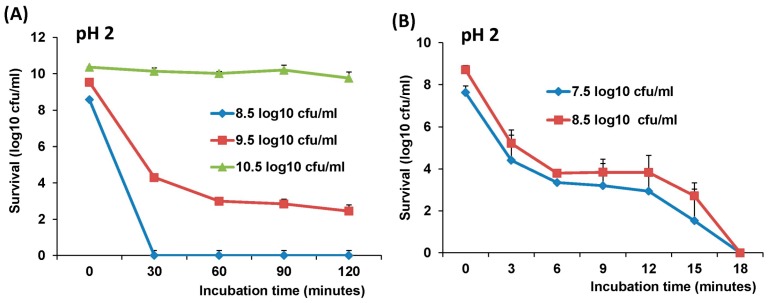
The effects of bacteria concentration and pH 2 on survival of LAB-VP1. (**A**) The survival of LAB-VP1 concentrated 10×, undiluted, and diluted 1:10 after placed in pH 2 saline solution. The tubes of each concentration were removed at 30, 60, 90, and 120 m. (**B**) The survival of LAB-VP1, diluted 1:10 and diluted 1:100 after being placed in pH 2 saline solution. The tubes of each concentration were removed at 3, 6, 9, 12, 15, and 18 m.

Finally, we determined the stability of LAB-VP1 in stomach pH. LAB-VP1 was placed in pH 2 saline to assess the survival after 2 h incubation. For undiluted LAB-VP1, 5.2 log reductions were detected after 30 min incubation, and 6.5–7.1 log reductions were observed after 60–120 min incubation (Figure 6A). No bacterial survival (8.9 log bacteria reductions) was detected when LAB-VP1 was diluted 10 times after 30 min incubation at pH 2.0. Interestingly, there was no significant titer reduction when LAB-VP1 was concentrated 10 times and incubated at pH 2 for 2h (*p* > 0.05). This suggests that a higher concentration of LAB-VP1 would enhance survival in stomach acid environment. Since all bacteria were inactivated at pH 2.0 after 30 min incubation when LAB-VP1 was diluted 1:10, we further determined the bacterial survival by reducing the incubation time (3, 6, 12, 15, and 18 min). As shown in Figure 6B, both 1:10 and 1:100 diluted LAB-VP1 were inactivated after 18 min incubation. Samples from 1:10 dilution had less bacteria reduction compared to those from 1:100 dilution, although there was no significant difference between these two groups (*p* > 0.05), with the exception of samples at 6 min incubation (*p* < 0.05).

### 3.6. Direct Comparison of Survival of LAB-VP1 in Various pH

The above results suggest that the survival of LAB-VP1 is enhanced at a higher pH. Next, we directly compared the survival of LAB-VP1 among a range of pH solutions. The pH stability assays were described previously with the exception of incubation time changing to 12, 24, 36, and 48 h.

**Figure 7:**
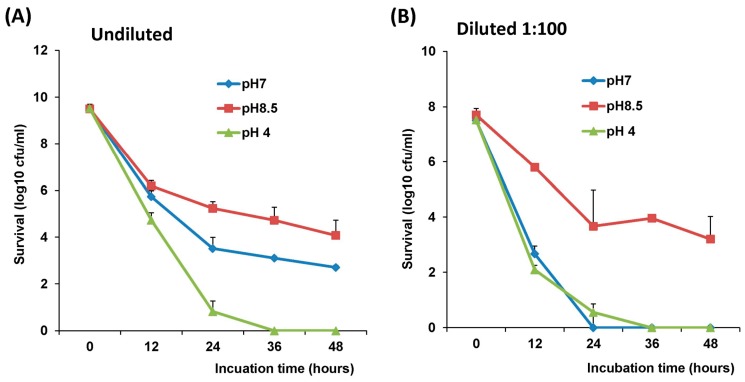
Direct comparison of the stability of LAB-VP1 in various pH saline solutions. (**A**) The effects of pH on the stability of LAB-VP1 at a concentration of 10^7^ cfu/mL. The original LAB-VP1 was diluted 100 times (1:100), and was placed in pH 4, 7, and 8.5 saline solutions. The tubes of each concentration were removed at 12, 24, 36, and 48 h. (**B**) The effects of pH on the stability of LAB-VP1 at a concentration of 10^9^ cfu/mL. The undiluted LAB-VP1 (concentration of 10^9^ cfu/mL) were placed in pH 4, 7, and 8.5 saline solutions. The tubes of each concentration were removed at 12, 24, 36, and 48 h.

We first directly compared the survival of the undiluted LAB-VP1 (at concentration of 10^9^ cfu) at pH 8.5, 7.0, and 4.0. As shown in Figure 7A, the stability of LAB-VP1 in different pH environments can be ranked as such: pH 8.5 > pH 7.0 > pH 4.0. A pH of 4.0 is the most unstable environment for LAB-VP1, at which no bacteria survived after 36 h incubation. Next, we compared the stability of LAB-VP1 when they were diluted 100 times (concentration of 10^7^ cfu) (Figure 7B). The survival rate at pH 8.5 was significantly higher than those at pH 4 and pH 7 (*p* < 0.05). There was no significant difference between the pH 4 and pH 7 survival (*p* > 0.05). Together, these results demonstrated that the survival of LAB-VP1 is significantly enhanced in basic pH environments.

### 3.7. The Survival of LAB-VP1 in Storage at 4 °C

**Figure 8:**
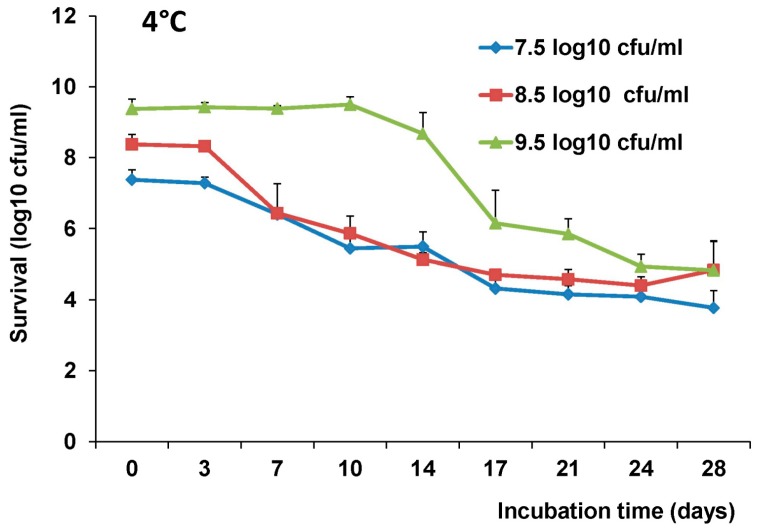
The stability of LAB-VP1 stored in 4 °C. The survival of LAB-VP1 stored in 4 °C. The concentrated 10×, undiluted, diluted 1:10, and diluted 1:100 LAB-VP1 was placed in pH 7 saline in 4 °C at 3, 7, 10, 14, 17, 21, 24, and 28 day time points, and bacterial survival was determined.

The stability of LAB-VP1 stored at 4 °C was assessed to determine the survival with cold storage for multiple weeks. Briefly, undiluted, diluted 1:10, and diluted 1:100 LAB-VP1 samples were incubated in pH 7 saline at 4 °C for 3, 7, 10, 14, 17, 21, 24, and 28 days, and the survival of LAB-VP1 was determined by bacterial counts (Figure 8). The undiluted LAB-VP1 was significantly more stable than the diluted samples for the first 10 days, then the log reduction started to increase. There was an increase in log reduction from day 14 to day 17 in the undiluted samples as the titer dropped close to the diluted titers. Overall, diluted 1:10 had a better survival rate than diluted 1:100, although there was no significant difference at some time points. Interestingly, all three concentrations ended with comparable titers after 28 days of incubation (Figure 8). This suggests that storage of LAB-VP1 in cold environments significantly enhances the stability of bacteria, and such stability of LAB-VP1 is concentration-dependent.

### 3.8. Fecal Shedding of LAB in Gnotobiotic Piglets

**Figure 9:**
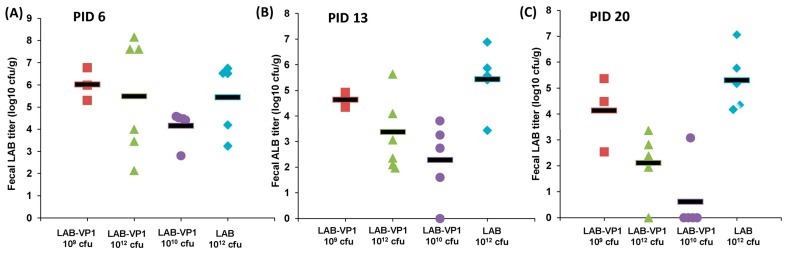
LAB shedding in piglet feces after oral vaccination. (**A**) LAB titer in piglet feces collected on PID 6. (**B**) LAB titer in piglet feces collected on PID 13. (**C**) LAB titer in piglet feces collected on PID 20. The group corresponding to different shapes is indicated in X axis.

Gnotobiotic piglets have been used as vaccination and challenge models for HuNoV since 2006 [28]. In this study, we determined whether oral delivery of LAB can be colonized in the pig intestine and whether LAB-based HuNoV vaccine is capable of protecting gnotobiotic piglets from HuNoV challenge. Experimental design and group information were summarized in Figure 1. For these experiments, there were 4 different groups of piglets, including LAB-VP1 10^9^ cfu, LAB-VP1 10^12^ cfu, LAB-VP1 10^10^ cfu, and LAB-empty vector 10^12^ cfu. The vaccine candidates were administered via oral gavage to 4-day-old piglets. After vaccination, fecal material was collected on post-inoculation day (PID) 6, PID 13, and PID 20 to determine the shedding of LAB in feces. The fecal LAB titer represents the colonization and growth of LAB in the intestines. The piglets were monitored daily following vaccination and there were no adverse reactions observed for the entirety of the experiment. LAB is a probiotic and is a generally recognized as safe (GRAS) agent, therefore no abnormal reaction was expected following the vaccination. The groups all had comparable amounts of LAB shed in fecal samples collected on PID 6, as shown in Figure 9A (*p* > 0.05). The concentrations ranged from 10^8.1^ to 10^2.1^ cfu/g feces.

The fecal shedding continued at PID 13 in the majority of the pigs (Figure 9B). There was no significant difference among these groups (*p* > 0.05). The LAB concentration ranged from 10^2^ to 10^7.4^ cfu/g feces, which is similar to the range observed in PID 6. There was a piglet from the LAB-VP1 10^10^ group that did not have detectable amount of LAB shedding. Fecal LAB was still detectable at day 20 post-vaccination (Figure 9C). The LAB-VP1 concentration ranged from 10^2^ to 10^5.5^ cfu/g feces, while LAB-empty vector concentration ranged from 10^4^ to 10^7^ cfu/g feces. These results indicate that LAB shedding in feces continued for at least 3 weeks in the majority of piglets.

### 3.9. LAB Titer in the Piglet Intestine and Colon

**Figure 10:**
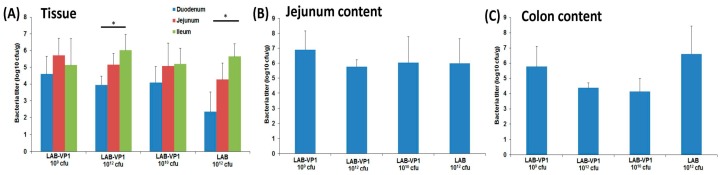
LAB titer in piglet intestine and colon content at necropsy. (**A**) LAB titer in piglet intestine sections on PCD 5. (**B**) LAB titer in the piglet jejunum content on PCD 5. (**C**) LAB titer in the piglet colon content on PCD 5. Data are expressed as the means ± standard deviations for each group.

At day 21 post-vaccination, all piglets were challenged with 10^7^ RNA genome copies of HuNoV. At day 5 post-challenge, all piglets were euthanized. During necropsy, intestine sections (duodenum, jejunum, and ileum), spleen, mesenteric lymph nodes, jejunum content, colon content, and blood were collected from each piglet. To determine the amount of LAB present in the intestines, the intestinal tissue pieces were scraped with a scalpel, eluted in saline, and the LAB titer was determined by plate count (Figure 10). As shown in Figure 10A, the ileum had significantly more LAB than the duodenum in the LAB-VP1 10^10^ cfu group and LAB 10^12^ cfu groups. In all the groups, the highest titer was in the ileum, while the lowest was in the duodenum. Among all the groups, the duodenum titers range from 10^2.4^ to 10^4.5^ cfu/g tissue, the jejunum titers from 10^4^ to 10^5.5^ cfu/g tissue, and the ileum from 10^5^ to 10^6^ cfu/g tissue. The content of the jejunum was collected to determine the amount of LAB. It was found that the LAB concentration was comparable between groups (*p* > 0.05), ranging from 10^5^ to 10^7^ cfu/g content (Figure 10B). Next, we determined the LAB titer in colon content. The concentration of LAB ranged from 10^4^ to 10^6.6^ cfu/g feces and was comparable among all the groups (*p* > 0.05) (Figure 10C). Taken together, a significant amount of LAB was detected in different sections of the intestine, jejunum content, and colon content of all groups. These results suggest that LAB may be capable of multiplying in pig intestines and surviving for at least 25 days.

### 3.10. Presence of LAB in Spleen and Mesenteric Lymph Nodes

Next, we determined whether LAB can be found in non-gastrointestinal tissue, including the spleen and mesenteric lymph nodes. Briefly, the spleen and mesenteric lymph nodes were isolated aseptically, homogenized with saline, and plated to determine the live LAB titer. No live LAB was detected in spleen or mesenteric lymph node tissues of all groups.

**Figure 11:**
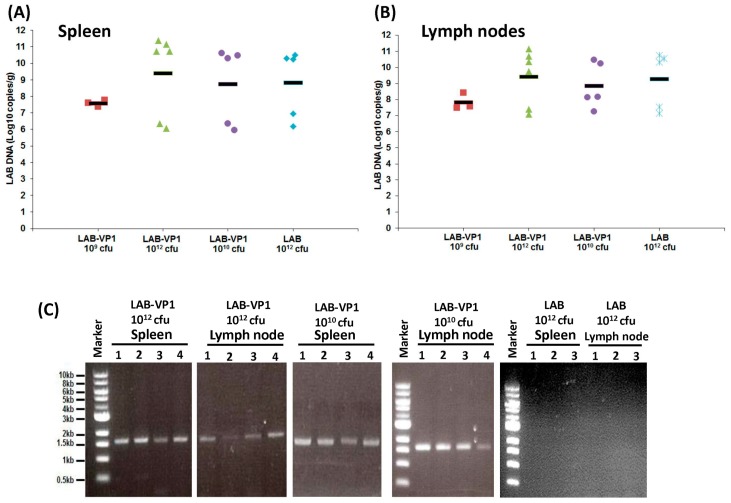
Presence of LAB DNA in mesenteric lymph nodes and spleen of gnotobiotic piglets. (**A**) LAB DNA copies in spleen tissue at PCD 5 determined by real-time PCR. The group corresponding to different shapes is indicated in X axis. (**B**) LAB DNA copies in the mesenteric lymph node tissue at PCD 5 determined by real-time PCR. (**C**) Detection of HuNoV VP1 gene in spleen and lymph nodes by PCR from selected groups.

Since live LAB was not detectable in lymph node and spleen tissue, we next determined whether LAB DNA presents these tissues. To do this, total DNA was extracted from the homogenized spleen and mesenteric lymph nodes, and the LAB DNA was quantified by real-time qPCR using primers annealing to the plasmid in the LAB. As shown in Figure 11A,B, high amounts of LAB DNA copies were detected in both spleen and mesenteric lymph nodes. However, the LAB DNA copies/g tissue in the spleen were similar among all the groups (*p* > 0.05), ranging from 6–11 log_10_ DNA copies/g tissue. Similarly, no significant difference in LAB DNA copies was observed in the mesenteric lymph nodes among all groups (*p* > 0.05). The presence of LAB DNA in the spleen and mesenteric lymph nodes suggests the capture of LAB by immune cells, and subsequent migration to these immune organs.

Since high copies of LAB DNA were detected in spleen and mesenteric lymph nodes, we next assessed whether these LAB were still carrying the VP1 gene. To do this, a traditional PCR was carried out using two primers annealing to VP1 gene. As shown in Figure 11C, spleen and mesenteric lymph nodes from LAB-VP1 groups, but not LAB vector control group, had detectable VP1 genes. This suggested that LAB-VP1 retained the VP1 gene, despite having been migrated into the spleen and mesenteric lymph nodes.

### 3.11. Oral Vaccination of LAB-Based Vaccine Induced HuNoV-Specific IgG Response

**Figure 12:**
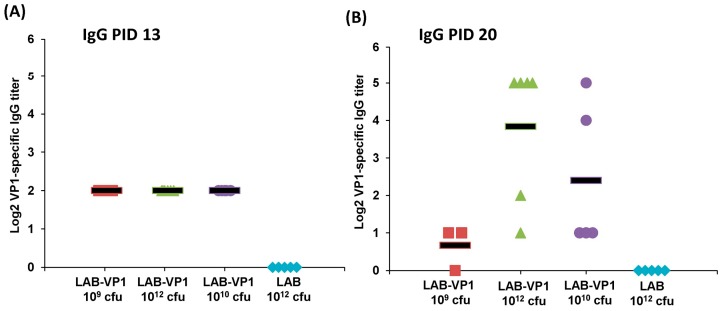
HuNoV-specific IgG response from oral vaccination of LAB-VP1 in gnotobiotic piglets. (**A**) The titer of HuNoV-specific IgG on PID13 measured with ELISA. (**B**) The titer of HuNoV-specific IgG on PID20 measured with ELISA. The group corresponding to different shapes is indicated in X axis.

To determine whether a LAB-based vaccine will trigger HuNoV-specific IgG responses, serum was isolated from each piglet at days 13 and 20 post-vaccination, and HuNoV-specific IgG titer was detected by ELISA. As shown in Figure 12A, HuNoV-specific IgG was detectable in all LAB-based HuNoV vaccine groups, except the LAB control group, at 13 days post-vaccination. However, there was no significant difference in HuNoV-specific IgG levels among vaccine groups containing LAB-VP1 (*p* > 0.05). At day 20 post-vaccination, HuNoV-specific IgG titer was increased in LAB-VP1 10^12^ cfu group (*p* < 0.05) (Figure 12B). However, the average IgG titer in other groups at day 20 was similar to that of day 13 (*p* > 0.05). This result demonstrates that the LAB-based HuNoV vaccine triggered a HuNoV-specific IgG response in gnotobiotic piglets.

### 3.12. Oral Vaccination of LAB-based Vaccine Induced HuNoV-Specific IgA Response

**Figure 13:**
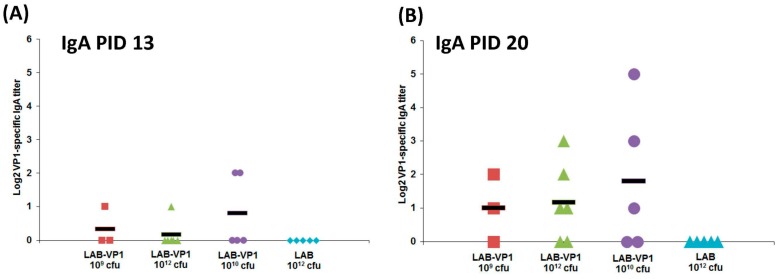
HuNoV-specific IgA response from oral vaccination of LAB-VP1 in gnotobiotic piglets. (**A**) The titer of HuNoV-specific of IgA on PID13 measured with ELISA. (**B**) The titer of HuNoV-specific IgG on PID20 measured with ELISA.

To determine whether a LAB-based HuNoV vaccine triggers a HuNoV-specific IgA response, feces were collected from each piglet at days 13 and 20 post-vaccination, and HuNoV-specific IgA responses were detected by ELISA. As shown in Figure 13A, HuNoV-specific IgA antibody was detectable in some of the piglets vaccinated with a vaccine containing LAB-VP1. One piglet from the LAB-VP1 10^9^ cfu group, one piglet from the LAB-VP1 10^12^ cfu group, and two piglets from LAB-VP1 10^10^ group had detectable IgA. However, none of piglets in the LAB control group had detectable HuNoV-specific IgA. At day 20 post-vaccination, more piglets developed HuNoV-specific IgA (Figure 13B). Overall, 3 out of 5 piglets in the LAB-VP1 10^10^ cfu group, 3 out 6 piglets in the LAB-VP1 10^12^ cfu group, and 2 out of 3 piglets in the LAB-VP1 10^9^ cfu group were positive for HuNoV-specific IgA. There were no significant differences among these groups (*p* > 0.05). In contrast, none of the piglets in the LAB control group were positive for HuNoV-specific IgA. Therefore, the LAB-based HuNoV vaccine triggered a HuNoV-specific IgA antibody response.

### 3.13. HuNoV Shedding Following Challenge in Gnotobiotic Piglets

**Figure 14:**
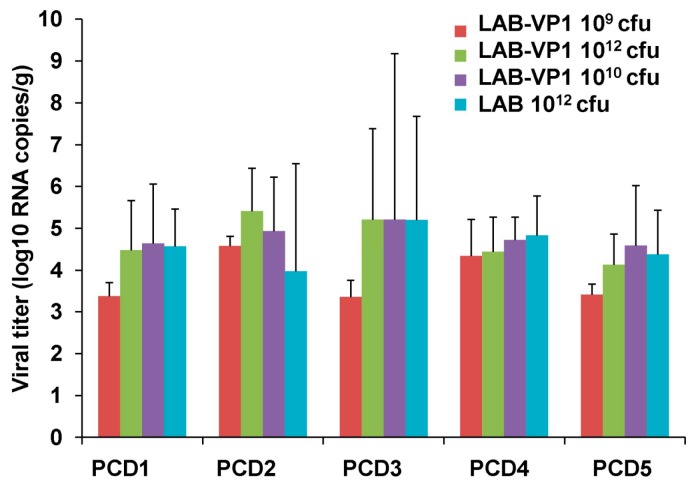
HuNoV RNA titer in feces. The fecal samples were collected from each piglet until PID 5 after challenge with HuNoV. Total RNA was extracted, and the HuNoV RNA was quantified by real-time-PCR.

To determine whether a LAB-based HuNoV vaccine protects gnotobiotic piglets from HuNoV shedding, fecal samples were collected from each piglet at days 1–5 after challenge with HuNoV GII.4 strain. Total RNA was extracted from fecal samples, and HuNoV RNA was quantified by real-time RT-PCR. The presence and average titer of viral RNA detected in pig feces at each PID are summarized in Figure 14. There was no significant difference in HuNoV shedding among all groups (*p* > 0.05), including the LAB vector control group.

### 3.14. LAB-based HuNoV Vaccine Prevented HuNoV Antigen Expression in Small Intestines of Gnotobiotic Piglets Following Challenge

**Figure 15:**
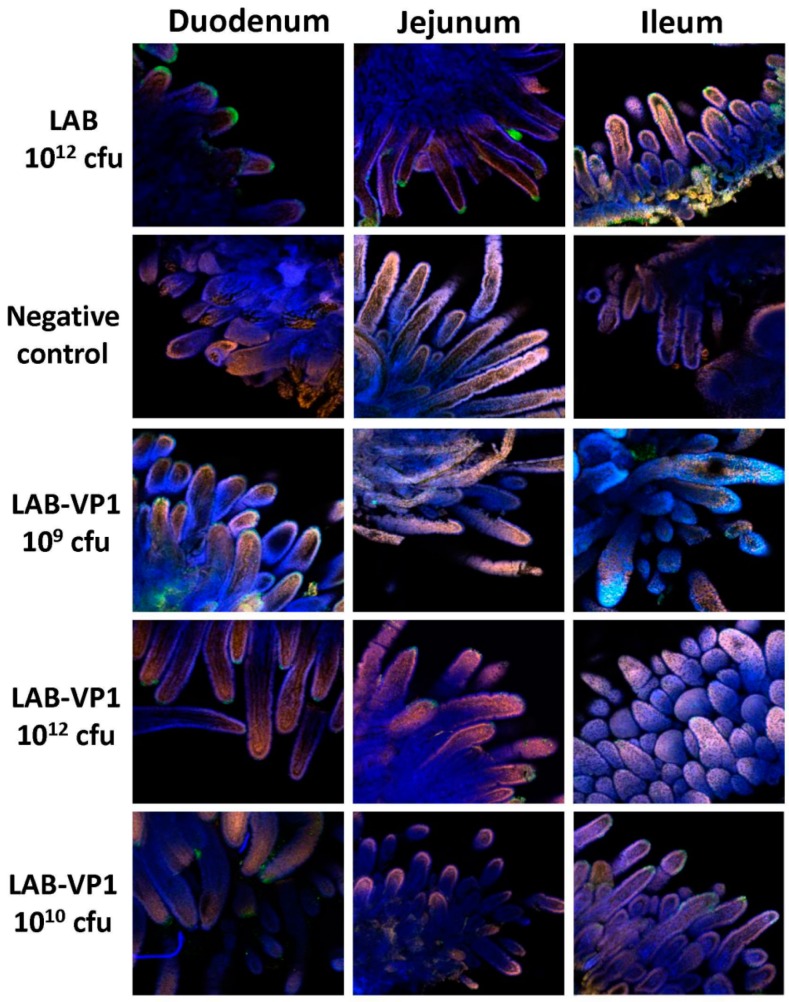
Detection of HuNoV antigen-positive cells in the intestine by IFA. Fresh intestinal tissues collected from inoculated piglets at PID 5 were sectioned into small pieces, fixed, quenched, and permeabilized. The tissues were incubated with HuNoV VP1-specific polyclonal antibody, followed by incubation with goat anti-guinea pig IgG labeled with Alexa Fluor 488 antibody. Nuclei and actin were counterstained with SYTOX orange and phalloidin labeled with Alexa Fluor 633. The stained tissues were mounted onto slides and examined using an Olympus FV1000 confocal microscopy imaging system. Representative microscope images (10× magnifications) from each group were shown. The size of villus may be variable because of the individual difference of the piglets. A green signal represents HuNoV antigen, a blue signal is actin, and an orange signal is the nucleus.

Finally, we determined whether HuNoV antigens could be detected in intestinal tissues after challenge. To do this, fresh duodenum, jejunum, and ileum tissues were collected at PID 5 and subjected to a whole-mount tissue indirect immunofluorescence assay (IFA) using a polyclonal antibody against the VP1 protein of HuNoV. The presence of HuNoV VP1 antigens was visualized by confocal fluorescence microscopy. As shown in Table 1 and Figure 15, a large number of HuNoV-positive staining (green) cells in duodenum and jejunum tissues from LAB control-vaccinated gnotobiotic piglets were detected. The HuNoV positive staining of cells at villous tips and the adjacent sides of individual villi indicate that the replication of HuNoV occurred in enterocytes, and that HuNoV antigens were expressed in enterocytes in these cells. In contrast, significantly fewer HuNoV-positive staining (green) cells were observed in all LAB-based vaccine groups. No antigen expression was detected in the negative control (LAB group without anti-HuNoV antibody). These data suggest that LAB-based HuNoV either diminished HuNoV antigen expression and possibly viral replication in pig intestine.

## 4. Discussion

There is a need for a safe and efficacious HuNoV vaccine to prevent and control the spread of HuNoV. LAB-based vaccine candidates have been developed for a number of viruses, bacteria, and parasites [29,30]. However, whether LAB can be used as a vector to deliver HuNoV has not been explored. In this study, we developed a LAB-based HuNoV vaccine candidate. We first showed that HuNoV VP1 was highly expressed in bacteria cells and can be secreted into culture supernatant. The stability of LAB-VP1 was influenced by multiple factors, such as pH, storage temperature, and bacteria concentration. The stability of HuNoV VP1 was enhanced in an alkaline pH (8.5), at a lower temperature (4 °C), and at a higher concentration. Finally, we showed that oral vaccination by the LAB-based HuNoV vaccine triggered HuNoV-specific IgA and IgG responses and diminished HuNoV replication in a novel gnotobiotic pig model. Our data indicates that LAB-based HuNoV is a promising vaccine candidate for HuNoV.

### 4.1. The Stability of LAB-VP1 in Different pH Environments

The survival of LAB-VP1 in the GI tract has been a challenge due to the harsh acidic stomach acid and alkaline environment in the duodenum. In addition, the enzymes secreted in the digestive tract will likely impact the stability of LAB-VP1. The pH of saliva is usually between 6.5–7.5. In the stomach, the pH reaches 1.5–2.5. After mixing food and stomach juices, it then enters the duodenum of the small intestine, where the pH raises to 7.0–8.5. We determined the stability of LAB-VP1 in different pH environments corresponding to the different GI tract regions. We demonstrated that LAB-VP1 was significantly more stable when starting in basic solutions (pH 8.5) than neutral and acidic solutions pH (*p* < 0.05). Incubation of undiluted LAB-VP1 at pH 8.5 at 37 °C for 48 h resulted in a 5.6 log reduction, whereas pH 7.0 and 4.0 led to 6.5 and 9.5 log reductions, respectively. At pH 2.0, the undiluted LAB-VP1 had a 5.2 log reduction after 30 min incubation. These results demonstrate that LAB-VP1 is highly susceptible to acidic pH environment.

The most convenient and inexpensive cold storage for bacteria is 4 °C. As expected, we showed that LAB-VP1 had a much better survival rate in 4 °C than 37 °C. The LAB metabolic processes slow down when the temperature drops, thus allowing for prolonged survival. When LAB (*Lactobacillus gasseri*) was stored in 4 °C, the bacteria survived for 28 days, as was observed with LAB-VP1 [31]. The bacteria carrying VP1 remains stable after storage in 4 °C for 42 days or −80 °C (in glycerol) for at least three years, as determined by PCR to detect VP1 gene and Western blot to determine VP1 expression.

We also found that bacteria concentration affected the survival of LAB-VP1 in stressful conditions. In all cases, a higher concentration of LAB-VP1 in solutions enhanced the survival of bacteria, whereas lower concentrations of LAB-VP1 reduced the survival rate. It is known that *Lactobacillus* uses the cell density-dependent quorum sensing system to regulate the expression of related genes to make themselves more adaptable to the surrounding environment [32]. It is likely that higher densities of bacterial cells would have higher quorum sensing activity levels, enhancing cell-cell communication and increasing survival rates. In addition, a higher concentration of LAB may produce higher amount of metabolites, such as lactic acid, sugar, and other small molecules, which protect the bacteria from autolysis. Difference in bacterial strains is another factor that influences the stability of LAB in acidic environments. When *L. lactis* is exposed to acid stress, oxidative stress proteins and heat shock proteins are expressed to help survival within the harsh acidic environment. It was shown that *Lactobacillus acidophilus* JCM 1132c and *Lactococcus lactis* subsp. *Lactis* bv. Diacetylactis N7 had 0.5 and 4.5 log reductions after treatment at pH 2.5 at 37 °C for 30 min, respectively, whereas *Lactococcus lactis* subsp. *Cremoris* (ATCC 19257) and *Lactococcus lactis* subsp. *Lactis* bv. Diacetylactis DRC1 were almost completely inactivated at the same conditions [32].

### 4.2. LAB-VP1 Shedding and Persistence in Gnotobiotic Piglets

Gnotobiotic piglets provide a good animal model to investigate the persistence of LAB-VP1 in the digestive system and evaluate LAB-VP1 shedding in their feces. These piglets are germ free, lack microflora, and share many similarities with humans in gastrointestinal structure, physiology, and immunology. The ability for LAB-VP1 to survive in the digestive tract is critical in stimulating an immune response. To evaluate the survival of LAB in the GI tract, newborn gnotobiotic piglets were orally inoculated with various doses, and fecal samples were taken to quantify LAB titer. We detected high LAB titers at days 6, 13, and 20 post-inoculation. Surprisingly, no significant differences were observed among the different doses of LAB-VP1, ranging from 10^9^ cfu to 10^12^ cfu per piglet (*p* > 0.05). At day 25, all piglets were euthanized. High amounts of LAB were detected in different sections of the small intestine, and in contents of the jejunum and colon. This demonstrates that LAB can survive in the pig digestive system and be shed in pig feces for at least 25 days. Although we are not able to demonstrate whether LAB-VP1 is capable of colonizing in the gut of the pig, it is clear that LAB survived the harsh acidic pH in the stomach and grew in the small and large intestine. For example, 10^5–7^ cfu/g feces of LAB-VP1 were detected in some piglets inoculated with 10^9^ cfu of LAB-VP1. Given that a normal piglet produces at least 100 g of feces per day, the amount of LAB-VP1 shedding far exceeds the input LAB-VP1. Thus, bacteria cells must have been multiplying in the pig digestive tracts. Although it is unclear why LAB-VP1 shedding was similar in all groups, it is possible that a dose of 10^9^ cfu is sufficient for enough bacteria to survive through the acidity of the stomach and subsequently multiply in the intestine. Further studies are needed to investigate the level of LAB shedding in gnotobiotic piglets at a dose lower than 10^9^ cfu per piglet.

Currently, it is still controversial whether LAB can colonize in the intestine after oral ingestion. However, it is believed that some LAB strains can establish colonization, for at least short-term, which may be sufficient to deliver and release a foreign antigen, stimulating an immune response. Although LAB has been used as a vector to deliver antigens of many pathogens, most of these studies used small animals (such as mice and rats) to investigate survival and colonization in the gut. LAB shedding after oral vaccination in mice varies from 12 h to 10 days [32,33]. In humans, it has been shown that oral administration of probiotic bacteria can produce temporary colonization of the intestine in patients with fully developed gut microflora. Several probiotics have been shown to be able to attach to the human intestinal mucosa. For example, oral administration of *Lactobacillus* GG into infants can result in a fecal recovery of the administered strain in feces 2 to 12-weeks post-administration [34]. This suggests that probiotics are capable of multiplying and surviving in the normal intestinal tract. Similarly, in our study, we found that a significant amount of LAB shed in the feces of the gnotobiotic piglets for at least 25 days. Future studies will be needed to determine how long LAB-VP1 can survive in gnotobiotic piglets.

### 4.3. Immunogenicity of LAB-Based HuNoV Vaccine in Gnotobiotic Piglets

Despite major efforts, there is no FDA approved HuNoV vaccine to protect people from HuNoV infection. This is due, in major part, to the fact that HuNoV lacks an efficient culture system and a small animal model to evaluate the efficacy of vaccine. To date, most HuNoV vaccine studies have used HuNoV virus-like particles (VLPs) as immunogen(s). Oral or intranasal immunization with VLPs induces variable humoral, mucosal, and cellular immunity [35,36]. The safety concern of viral vectored vaccine candidates (such as NDV, VEE, VSV, and adenovirus) prevented the clinical trials in humans. Although the VLP-based vaccine candidate is promising, there is a need to explore alternative strategies to develop a HuNoV vaccine.

An ideal HuNoV vaccine should be safe, stable, inexpensive, easy to deliver, and should induce robust humoral, mucosal, and cellular immune responses at sites where pathogens interact with the host. Therefore, this can generate immediate (innate) and long-term (acquired) immune barriers against infection. Although none of food grade LAB vectored vaccines have been used in humans, animal studies and preclinical trials showed that they may be promising vaccine candidates for future development. There are several advantages of using food grade LAB as a vector. First, LABs are natural probiotics, which are safe for human consumption. Second, LAB can survive passage through gastric acid and are able to grow in the gut, as well as provide long-term boost effects of target vaccines. Third, high levels of antigen expression can be achieved using LAB as the vehicle. Fourth, LAB-based vaccines can elicit both mucosal and systemic immune responses, which have been shown for many other pathogens. Fifth, LAB-expressed antigens can be absorbed into Peyer’s patches, the inductive sites of the mucosal immune system. Finally, LAB is easily grown and inexpensive, thus can facilitate the large-scale production of vaccines [37].

In our study, we showed that HuNoV VP1 can be highly expressed by a LAB vector. A large number of VP1 can be detected in bacterial cell lysates and supernatants. These VP1 proteins will likely be captured by antigen-presenting cells, which, in turn, generate HuNoV-specific immunity. Using a unique gnotobiotic piglet model, we showed that a LAB-based vaccine triggered HuNoV-specific fecal IgA and serum IgG responses as early as day 13 post-vaccination. This response was even greater at day 20 post-vaccination. Gnotobiotic piglets have previously been shown to be susceptible to HuNoV infection. However, the robustness of infection will depend on the strain of HuNoV, the inoculation level, age of the piglet, and the level of HBGA expression in the piglet’s intestine. It was shown that gnotobiotic piglets infected by some HuNoV strains can develop mild diarrhea and HuNoV shedding in feces [38,39]. Based on our previous study and others [38,39], HuNoV antigen expression can be detected in the small intestine but not the large intestine (such as cecum and colon). Within the small intestine, the duodenum typically had significantly more HuNoV expression than the jejunum and ileum (Table 1 and Figure 15). In our study, we did not observe a significant difference in HuNoV shedding in feces after the HuNoV challenge. Importantly, we found that the LAB control group had a large number of HuNoV antigen expression in the duodenum, and had relatively less HuNoV antigen expression in the jejunum and ileum section. Importantly, all groups that received LAB-based vaccines containing LAB-VP1 had either significantly less or no HuNoV expression in duodenum sections, and essentially no antigens were detected in the jejunum and ileum sections. These results demonstrate that the LAB-based HuNoV vaccine diminished HuNoV antigen expression and possibly viral replication in the small intestine.

Although we clearly showed LAB-VP1 is immunogenic, there are a few limitations in this study which need to be addressed in future study. We did not observe a significant difference in HuNoV shedding among groups after challenge with HuNoV, as quantified by real-time RT-PCR. Since real-time RT-PCR cannot discriminate infectious and non-infectious particles, it remains possible there are less infectious HuNoV in vaccinated group. This is supported by the observation that little HuNoV antigen expression was found in LAB-VP1-vaccinated groups after challenge. A cultivable HuNoV strain will be needed to address this question. In our study, we did not observe a dose effect for the LAB-based HuNoV vaccine. We used three doses of LAB-based vaccines for vaccination: 10^9^, 10^10^, and 10^12^ cfu per piglet. Unfortunately, we did not observe a significant difference in HuNoV-specific IgA and IgG responses between these groups. In this experiment, we only tested the efficacy of a single vaccination within three weeks after vaccination. Future experiments should examine the long-term immune responses, the effects of booster vaccination, and the effects of adjuvants. Live bacteria were negative for all groups in both the spleen and mesenteric lymph nodes, including the 10^12^ cfu group, which received a 1000 times higher dose of LAB-VP1. Interestingly, when LAB DNA in the spleen and lymph nodes was quantified by qPCR, all groups had similar levels of LAB DNA copies. This suggests that intestinal LAB can be captured by antigen-presenting cells. Future studies are needed to determine mechanisms of LAB-VP1-induced immune responses and protection.

In summary, we have constructed a LAB-based vaccine candidate for HuNoV that is immunogenic in gnotobiotic piglets. Further optimization of this innovative vaccine strategy will shed light on vaccine development for HuNoV and other non-cultivable food- and water-borne viruses.

## Figures and Tables

**Table 1:** Summary of IFA in pig intestine.

Group	Duodenum		Jejunum		Ileum	
	IFA Positive ^A^	Average Score ^B^	IFA Positive	Average Score	IFA Positive	Average Score
LAB-VP1 10^12^ cfu	1/6	0.167	3/6	0.50	1/6	0.167
LAB-VP1 10^10^ cfu	2/5	0.60	0/5	0	2/5	0.40
LAB-VP1 10^9^ cfu	2/3	1.333	1/3	0.333	1/3	0.333
LAB 10^12^ cfu control	5/5	2.60	5/5	2.00	5/5	1.40

Note: A indicates IFA positive piglets in total piglets in each group. B indicates average score of IFA signal in each group. IFA signal was scored for duodenum, jejunum, and ileum sections at ×10 magnification based on the following criteria: score 0 = no IFA signal was observed in villi; score 1 = a small amount of IFA signal was observed in villi; score 2 = a moderate level of IFA signal was observed in villi; and score 3 = a large amount of IFA signal was observed in villi. Average score for each group is shown.

## References

[1] Estes M.K., Prasad B.V., Atmar R.L. (2006). Noroviruses everywhere: Has something changed?. Curr. Opin. Infect. Dis..

[2] Bartsch S.M., Lopman B.A., Ozawa S., Hall A.J., Lee B.Y. (2016). Global economic burden of norovirus gastroenteritis. PLoS ONE.

[3] Belliot G., Lopman B.A., Ambert-Balay K., Pothier P. (2014). The burden of norovirus gastroenteritis: An important foodborne and healthcare-related infection. Clin. Microbiol. Infect..

[4] Caul E.O. (1994). Small round structured viruses: Airborne transmission and hospital control. Lancet.

[5] Donaldson E.F., Lindesmith L.C., Lobue A.D., Baric R.S. (2008). Norovirus pathogenesis: Mechanisms of persistence and immune evasion in human populations. Immunol. Rev..

[6] NIAID (2016). NIAID Emerging Infectious Diseases/Pathogens|NIH: National Institute Of Allergy And Infectious Diseases. https://www.niaid.nih.gov/research/emerging-infectious-diseases-pathogens.

[7] Ball J.M., Hardy M.E., Atmar R.L., Conner M.E., Estes M.K. (1998). Oral immunization with recombinant norwalk virus-like particles induces a systemic and mucosal immune response in mice. J. Virol..

[8] Bok K., Parra G.I., Mitra T., Abente E., Shaver C.K., Boon D., Engle R., Yu C., Kapikian A.Z., Sosnovtsev S.V. (2011). Chimpanzees as an animal model for human norovirus infection and vaccine development. Proc. Natl. Acad. Sci. USA.

[9] Jiang X., Wang M., Graham D.Y., Estes M.K. (1992). Expression, self-assembly, and antigenicity of the norwalk virus capsid protein. J. Virol..

[10] Souza M., Costantini V., Azevedo M.S., Saif L.J. (2007). A human norovirus-like particle vaccine adjuvanted with iscom or mlt induces cytokine and antibody responses and protection to the homologous GII.4 human norovirus in a gnotobiotic pig disease model. Vaccine.

[11] Ball J.M., Graham D.Y., Opekun A.R., Gilger M.A., Guerrero R.A., Estes M.K. (1999). Recombinant norwalk virus-like particles given orally to volunteers: Phase i study. Gastroenterology.

[12] Atmar R.L., Bernstein D.I., Harro C.D., Al-Ibrahim M.S., Chen W.H., Ferreira J., Estes M.K., Graham D.Y., Opekun A.R., Richardson C. (2011). Norovirus vaccine against experimental human norwalk virus illness. N. Engl. J. Med..

[13] Bernstein D.I., Atmar R.L., Lyon G.M., Treanor J.J., Chen W.H., Jiang X., Vinje J., Gregoricus N., Frenck R.W., Moe C.L. (2015). Norovirus vaccine against experimental human gii.4 virus illness: A challenge study in healthy adults. J. Infect. Dis..

[14] Guo L., Wang J., Zhou H., Si H., Wang M., Song J., Han B., Shu Y., Ren L., Qu J. (2008). Intranasal administration of a recombinant adenovirus expressing the norovirus capsid protein stimulates specific humoral, mucosal, and cellular immune responses in mice. Vaccine.

[15] Harrington P.R., Yount B., Johnston R.E., Davis N., Moe C., Baric R.S. (2002). Systemic, mucosal, and heterotypic immune induction in mice inoculated with venezuelan equine encephalitis replicons expressing norwalk virus-like particles. J. Virol..

[16] Kim S.H., Chen S., Jiang X., Green K.Y., Samal S.K. (2014). Newcastle disease virus vector producing human norovirus-like particles induces serum, cellular, and mucosal immune responses in mice. J. Virol..

[17] Ma Y., Li J. (2011). Vesicular stomatitis virus as a vector to deliver virus-like particles of human norovirus: A new vaccine candidate against an important noncultivable virus. J. Virol..

[18] Jones M.K., Watanabe M., Zhu S., Graves C.L., Keyes L.R., Grau K.R., Gonzalez-Hernandez M.B., Iovine N.M., Wobus C.E., Vinje J. (2014). Enteric bacteria promote human and mouse norovirus infection of b cells. Science.

[19] Lei S., Samuel H., Twitchell E., Bui T., Ramesh A., Wen K., Weiss M., Li G., Yang X., Jiang X. (2016). Enterobacter cloacae inhibits human norovirus infectivity in gnotobiotic pigs. Sci. Rep..

[20] Ettayebi K., Crawford S.E., Murakami K., Broughman J.R., Karandikar U., Tenge V.R., Neill F.H., Blutt S.E., Zeng X.L., Qu L. (2016). Replication of human noroviruses in stem cell-derived human enteroids. Science.

[21] Asensi G.F., de Sales N.F., Dutra F.F., Feijó D.F., Bozza M.T., Ulrich R.G., Miyoshi A., de Morais K., Azevedo V.A., Silva J.T. (2013). Oral immunization with lactococcus lactis secreting attenuated recombinant staphylococcal enterotoxin b induces a protective immune response in a murine model. Microb. Cell Fact..

[22] Cauchard S., Bermúdez-Humarán L.G., Blugeon S., Laugier C., Langella P., Cauchard J. (2011). Mucosal co-immunization of mice with recombinant lactococci secreting vapa antigen and leptin elicits a protective immune response against rhodococcus equi infection. Vaccine.

[23] Frankel F.R., Hegde S., Lieberman J., Paterson Y. (1995). Induction of cell-mediated immune responses to human immunodeficiency virus type 1 gag protein by using listeria monocytogenes as a live vaccine vector. J. Immunol..

[24] Hanniffy S.B., Carter A.T., Hitchin E., Wells J.M. (2007). Mucosal delivery of a pneumococcal vaccine using lactococcus lactis affords protection against respiratory infection. J. Infect. Dis..

[25] Miyoshi A., Poquet I., Azevedo V., Commissaire J., Bermudez-Humaran L., Domakova E., Le Loir Y., Oliveira S.C., Gruss A., Langella P. (2002). Controlled production of stable heterologous proteins in lactococcus lactis. Appl. Environ. Microbiol..

[26] Robinson K., Chamberlain L.M., Schofield K.M., Wells J.M., Le Page R.W. (1997). Oral vaccination of mice against tetanus with recombinant lactococcus lactis. Nat. Biotechnol..

[27] Wang K., Huang L., Kong J., Zhang X. (2008). Expression of the capsid protein of porcine circovirus type 2 in lactococcus lactis for oral vaccination. J. Virol. Methods.

[28] Cheetham S., Souza M., McGregor R., Meulia T., Wang Q., Saif L.J. (2007). Binding patterns of human norovirus-like particles to buccal and intestinal tissues of gnotobiotic pigs in relation to A/H histo-blood group antigen expression. J. Virol..

[29] Azizpour M., Hosseini S.D., Jafari P., Akbary N. (2017). A new strategy for vaccination. Avicenna. J. Med. Biotechnol..

[30] Wang M., Gao Z., Zhang Y., Pan L. (2016). Lactic acid bacteria as mucosal delivery vehicles: A realistic therapeutic option. Appl. Microbiol. Biotechnol..

[31] Chávarri M., Marañón I., Ares R., Ibáñez F.C., Marzo F., Villarán M.d.C. (2010). Microencapsulation of a probiotic and prebiotic in alginate-chitosan capsules improves survival in simulated gastro-intestinal conditions. Int. J. Food Microbiol..

[32] Kimoto H., Nomura M., Kobayashi M., Mizumachi K., Okamoto T. (2003). Survival of lactococci during passage through mouse digestive tract. Can. J. Microbiol..

[33] Pavan S., Desreumaux P., Mercenier A. (2003). Use of mouse models to evaluate the persistence, safety, and immune modulation capacities of lactic acid bacteria. Clin. Diagn. Lab. Immunol..

[34] Schultz M., Göttl C., Young R.J., Iwen P., Vanderhoof J.A. (2004). Administration of oral probiotic bacteria to pregnant women causes temporary infantile colonization. J. Pediatr. Gastroenterol. Nutr..

[35] Atmar R.L., Ramani S., Estes M.K. (2018). Human noroviruses: Recent advances in a 50-year history. Curr. Opin. Infect. Dis..

[36] Ramani S., Atmar R.L., Estes M.K. (2014). Epidemiology of human noroviruses and updates on vaccine development. Curr. Opin. Gastroenterol..

[37] Kim S.H., Jeung W., Choi I.D., Jeong J.W., Lee D.E., Huh C.S., Kim G.B., Hong S.S., Shim J.J., Lee J.L. (2016). Lactic acid bacteria improves peyer’s patch cell-mediated immunoglobulin a and tight-junction expression in a destructed gut microbial environment. J. Microbiol. Biotechnol..

[38] Lou F., Ye M., Ma Y., Li X., DiCaprio E., Chen H., Krakowka S., Hughes J., Kingsley D., Li J. (2015). A gnotobiotic pig model for determining human norovirus inactivation by high-pressure processing. Appl. Environ. Microbiol..

[39] Cheetham S., Souza M., Meulia T., Grimes S., Han M.G., Saif L.J. (2006). Pathogenesis of a genogroup ii human norovirus in gnotobiotic pigs. J. Virol..

